# Land Subsidence Susceptibility Mapping in South Korea Using Machine Learning Algorithms

**DOI:** 10.3390/s18082464

**Published:** 2018-07-30

**Authors:** Dieu Tien Bui, Himan Shahabi, Ataollah Shirzadi, Kamran Chapi, Biswajeet Pradhan, Wei Chen, Khabat Khosravi, Mahdi Panahi, Baharin Bin Ahmad, Lee Saro

**Affiliations:** 1 Geographic Information Science Research Group, Ton Duc Thang University, Ho Chi Minh City, Vietnam; buitiendieu@tdt.edu.vn; 2 Faculty of Environment and Labour Safety, Ton Duc Thang University, Ho Chi Minh City, Vietnam; 3 Department of Geomorphology, Faculty of Natural Resources, University of Kurdistan, Sanandaj 66177-15175, Iran; 4 Department of Rangeland and Watershed Management, Faculty of Natural Resources, University of Kurdistan, Sanandaj 66177-15175, Iran; a.shirzadi@uok.ac.ir (A.S.); k.chapi@uok.ac.ir (K.C.); 5 Centre for Advanced Modelling and Geospatial Information Systems (CAMGIS), Faculty of Engineering and IT, University of Technology Sydney, Sydney, NSW 2007, Australia; Biswajeet.Pradhan@uts.edu.au; 6 Department of Energy and Mineral Resources Engineering, Choongmu-gwan, Sejong University, 209 Neungdong-ro, Gwangjin-gu, Seoul 05006, Korea; 7 College of Geology & Environment, Xi’an University of Science and Technology, Xi’an 710054, China; chenwei.0930@163.com; 8 Department of Watershed Sciences Engineering, Faculty of Natural Resources, Sari Agricultural and Natural Resources University (SANRU), Sari, Mazandaran P.O.BOX 48181-68984, Iran; khabat.khosravi@gmail.com; 9 Department of Geophysics, Young Researchers and Elites Club, North Tehran Branch, Islamic Azad University, Tehran P.O. Box 19585/466, Iran; panahi2012@yahoo.com; 10 Department of Geoinformation, Faculty of Geoinformation and Real Estate, Universiti Teknologi Malaysia (UTM), Skudai 81310, Malaysia; baharinahmad@utm.my; 11 Geological Research Division, Korea Institute of Geoscience and Mineral Resources (KIGAM), 124, Gwahak-ro Yuseong-gu, Daejeon 34132, Korea; 12 Department of Geophysical Exploration, Korea University of Science and Technology, 217 Gajeong-ro Yuseong-gu, Daejeon 34113, Korea

**Keywords:** land subsidence, machine learning algorithms, GIS, South Korea

## Abstract

In this study, land subsidence susceptibility was assessed for a study area in South Korea by using four machine learning models including Bayesian Logistic Regression (BLR), Support Vector Machine (SVM), Logistic Model Tree (LMT) and Alternate Decision Tree (ADTree). Eight conditioning factors were distinguished as the most important affecting factors on land subsidence of Jeong-am area, including slope angle, distance to drift, drift density, geology, distance to lineament, lineament density, land use and rock-mass rating (RMR) were applied to modelling. About 24 previously occurred land subsidence were surveyed and used as training dataset (70% of data) and validation dataset (30% of data) in the modelling process. Each studied model generated a land subsidence susceptibility map (LSSM). The maps were verified using several appropriate tools including statistical indices, the area under the receiver operating characteristic (AUROC) and success rate (SR) and prediction rate (PR) curves. The results of this study indicated that the BLR model produced LSSM with higher acceptable accuracy and reliability compared to the other applied models, even though the other models also had reasonable results.

## 1. Introduction

Land subsidence is one of the land degradation features usually occur due to the diversity of natural or anthropic effects that cause a change in the environment and have social and economic effects [1]. Many land subsidence have occurred globally because of various reasons such as mining, dissolution of limestone, extraction of groundwater and natural gas, earthquake [2,3,4]. The land subsidence forms over a period of time due to overload above voids such as underground mining [5,6]. In South Korea, many land subsidence have occurred due to coal mining specifically in the 1960s and 1970s since the coal mining was playing an important role in the industry. In the 1980s, the coal mining industry was declined because the Korean government prepared an appropriate act to close and abandon most of coal mines [7].

Not only the abandoned mines did not decrease the environmental destructions including land subsidence and water pollution but also their risks were increased [8]. Especially, the underground land subsidence can create damage to surface structures, including house, building, railroad and roads, as well as human injury [5]. Since ground recovery after occurrence of a land subsidence is a challenge and also their rehabilitation is costly [6,9], cautionary operations and proper strategies for land subsidence are critical.

Basically, performing a successful land subsidence study is associated with considering an integration of several environmental related factors [7]. Therefore, a geo-database in land subsidence modelling must cover various types of thematic information such as geo-hydrological factors [10]. Remote sensing (RS) and Geographical information system (GIS) data are useful tools to integrate the development of the land subsidence studies [11,12]. On the other hand, accurate land subsidence inventories may still be challenging to acquire, although modern technologies such as Global Positioning Systems (GPS), RS and GIS may assist spatial prediction and localization of visible land subsidence features [13,14].

According to the literature overview, there are several models and methods (qualitative and quantitative) have been successfully applied and developed in different areas of the world as land subsidence susceptibility mapping (LSSM). The quantitative methods can deal with the disadvantages of qualitative ones which include: logistic regression (LR) [15], frequency ratio (FR) [16], analytical hierarchy processes (AHP) [17], weight-of-evidence (WOE) [18], evidential-belief-functions (EBF) [16], artificial neural network (ANN) [7], support vector machine (SVM) [19], random forest (RF), grey model (GM) [20], sensitivity analysis (SA) [6], fuzzy logic (FL) [21] and adaptive neuro-fuzzy inference system (ANFIS) [10].

Although some methods and techniques have been developed for preparing the LSSM, it seems that more logical and accurate results can be obtained by applying and comparing different methods. Therefore, single-based classifiers generally have less prediction accuracy rather than the ensemble models [22]. Basically, machine learning ensembles models have recently increased the performance and prediction accuracy of single-based classifiers [23]. The main advantage of machine learning algorithms (MLAs) is their ability to discover a complicated relationship in data, which is often unpredictable. Additionally, the MLAs can deal with spatial peculiarities of data patterns at various scales [24].

Application of data mining approaches to LSSM is very limited despite of their all advantageous. Therefore, these methods and techniques can still be investigated and compared with conventional methods to acquire an adequate background to reach reasonable conclusions for LSSM. Therefore, this study aimed to predict and map land subsidence by producing LSSM of a region in the vicinity of abandoned underground coal mines of South Korea by four commonly introduced machine learning algorithms including Bayesian Logistic Regression (BLR), Support Vector Machine (SVM), Logistic Model Tree (LMT) and Alternate Decision Tree (ADTree) methods. The reliability and prediction power (accuracy) of all the models were evaluated by the area under the ROC curve (AUROC), success rate (SR), prediction rate (PR), Freidman and Wilcoxon rank statistical tests. Data processing was conducted using ArcGIS 10.3 and also four machine learning algorithms were produced by WEKA 3.9.2 software.

## 2. Data Acquisition

### 2.1. Description of the Study Area

**Figure 1:**
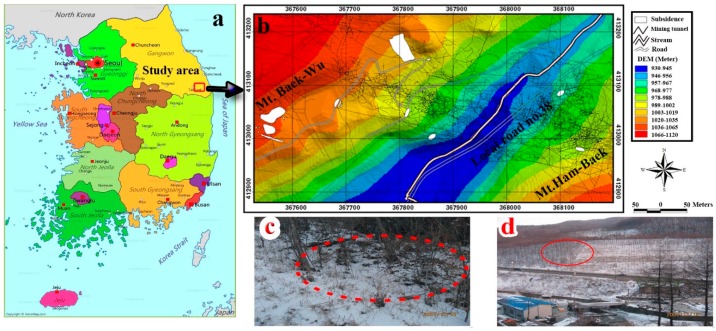
Study area; (**a**) Geographic location of the study area in the northeast of South Korea; (**b**) Location of study area between Mt. Baek-Wu to the west and Mt. Ham-Beak to the southeast; (**c**) and (**d**) the pictures at the surveyed subsidence locations that were taken from field surveys.

The study area, Jeong-am in South Korea is located with geographical position of 37°12′0″ and 37°13′0″ N in latitude and 128°53′10″–128°54′10″ E in longitude (Figure 1). The study area was a major coal mining area and has many cavities produced due to coal mining [7]. The geology of the study area consists of Jangseong and Hambaeksan Formations. The majority of South Korean coals (Jangseong Formation) has been accumulated in the upper Paleozoic and the lower Mesozoic eras [25]. This formation contains several thick coal beds [13] consisting of alternate layers of sandstone and shale which its shale layers have intercalations of two to three coal bed seams [22].

The coal mining in the study area occurred from 1967 until 1989. The average thickness of the coal seams was 1.3–2.5 m with rich seams reached 4–15 m in steep slopes (60°–70°) areas [22]. The trend of the abandoned drifts is to deepen from the center to the northeastern part along the direction of the Jangseong Formation dip in the study area. Also, the drifts are range from 70 to 260 m in depth [22].

Severe land subsidence has occurred in mountainous areas. A local road (No. 38) shows shape of typical sinkhole with deformations and cracks on the road [7]. The total area of land subsidence is 3296 m^2^ in the study area. The land subsidence locations are shown on topographic map (Figure 1).

### 2.2. Data Collection and Preparation

#### 2.2.1. Land Subsidence Inventory

Land subsidence inventory maps were prepared using various sources: with the help of satellite image interpretation (IKONOS), 1:5000 land subsidence map from Coal Industry Promotion Board, a 1:5000 topographic map from the National Geographic Information Institute (NGII), a 1:50,000 geological map from the Korea Institute of Geoscience and Mineral Resources (KIGAM), a 1:5000 land-use map from the NGII, a 1:1200 mine-tunnel map from Coal Industry Promotion Board and borehole data from the Coal Industry Promotion Board (1996) [22]. The maps show the locations of land subsidence in the study area. These maps generally help the prediction of locations and conditions of future land subsidence.

According to Coal Industry Promotion Board (1996), a total of 24 land subsidence are occurred with the average coal-seam thickness of 1–1.5 m which they cover an area about 3296 m^2^ [22]. In the present study area, a total number of 25 land subsidence locations were recognized (March 2015), randomly divided into 70% (17 land subsidence) as the training dataset and 30% (8 land subsidence) as the validation dataset. A land subsidence inventory map was produced by ArcGIS software.

#### 2.2.2. Land Subsidence Conditioning Factors

There are many important factors that contribute to land subsidence around coal mines. According to existing literature [5,6,7] and analysis on the study area, eight land subsidence conditioning factors were adopted in this study that includes slope angle, distance to drift, drift density, geology, distance to lineament, lineament density, land use and rock-mass rating (RMR). All the factors mentioned above were extracted from a digital elevation model (DEM), topographical and geological maps in a grid format with spatial resolution of 2 m × 2 m cells in 179 rows and 361 columns; the entire study area comprised 63,677 cells and ground subsidence had occurred in 824 cells. Reliable accuracy of the spatial database is indispensable in a GIS environment. For this reason, accurate maps authorized by national organizations such as the Coal Industry Promotion Board for ground subsidence, the National Geographic Information Institute for topography and land use, the Mine Reclamation Cororation. For mine tunnels and boreholes and the Korea Institute of Geoscience and Mineral Resources for geology were assembled even though the scales of the maps differed. All of scale factors except geology and land use were reclassified into five classes based on equal area using ESRI ArcGIS 10.3 for the probability analysis of the area of existing ground subsidence. Thus, the range of each class is automatically determined based on equal area.

The slope angle is an important factor in the assessment of land subsidence for current study and was extracted from the DEM with spatial resolution of 1 × 1 m. The slope angle factor was constructed with five categories: (1) 0–10; (2) 10–20; (3) 20–30; (4) 30–40; and (5) >40° (Table 1). In the present study, the 3-D digital map of drifts provided by Coal Industry Promotion Board of South Korea was converted to a grid file and then subtracted from the DEM for computing drift depth. Then, the distance from each drift was calculated using a proximity analysis for extraction of distance to drift (m) factor in five classes including (1) 0–2; (2) 2–8; (3) 8–19; (4) 19–50; and (5) >50 (Table 1). Drift density is another important conditioning factor in the occurrence of land subsidence. It is defined as the total length of all the drifts in a region divided by the total area of the region. Hence, an underground drift density factor was prepared using drift depth and the horizontal area of influence [21] in five categories (1) 0–0.002; (2) 0.002–0.0448; (3) 0.0448–0.120; (4) 0.120–0.299; and (5) 0.299–0.952 m/m^2^ (Table 1).

Geology effectively influences the occurrence of land subsidence especially in coal mining areas [5]. The location of the occurred subsidence in the study area is in a direct relationship with structures of geology and mining area complex [18]. A surface geology factor was extracted using the digital geological map with 1:50,000 scale issued by the KIGAM in two categories including (1) Gobangsan Group; and (2) Sadong Group (Table 1).

The spatial distribution of the lineaments in coal mining area is a considerable factor in the occurrence of land subsidence [5]. The lineaments of the study area were identified with a multispectral IKONOS-PAN-sharpened image (with 1 m resolution, ortho-rectified) of a mine area, which was captured in October 2010. A map showing 1-m interval distances to lineament was computed by the Euclidean distance method in ArcGIS 10.3 in five categories including (1) 0–10; (2) 10–20; (3) 20–30; (4) 30–60; and (5) >60 m. Also, lineament density factor was constructed with five categories: (1) 0–0.001; (2) 0.001–0.029; (3) 0.029–0.0435; (4) 0.0435–0.052; and (5) 0.052–0.109 m/m^2^ (Table 1). 

Land use is another factor in the evaluation of the occurred land subsidence in the study area was obtained from a digital land characteristics map supplied in a grid format with spatial resolution of 1 m × 1 m by the NGII in the drawing exchange file (DXF) format in in nine classes including (1) mixed forest lands; (2) deciduous forests; (3) mixed barren lands; (4) commercial areas; (5) coniferous forests; (6) other grasses; (7) transportation; (8) natural grasses; and (9) fields (Table 1).

RMR, as a geomechanical rock classification system which developed between 1972 and 1973 [26], was used for the study area based on several parameters including the uniaxial compressive strength of rock material, rock quality designation, spacing of discontinuities, condition of discontinuities and groundwater conditions [27]. An inverse-distance weighted (IDW) interpolation was used to contour the RMR [7]. The RMR factor was classified into five categories including (1) 0.00366–1.26; (2) 1.26–1.54; (3) 1.54–1.93; (4) 1.93–2.79; and (5) 2.79–4 (Table 1).

## 3. Methodology

### 3.1. Background of Machine Learning Algorithms

**Figure 2:**
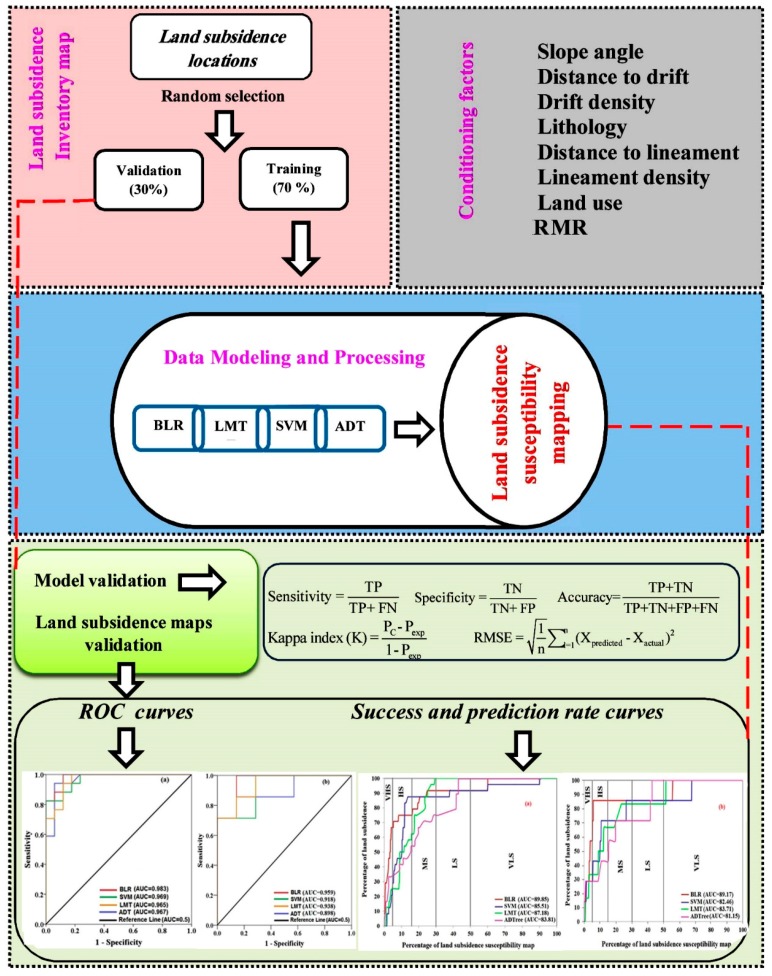
The flowchart of land subsidence modelling process in the study area.

The following steps were performed to prepare the land subsidence susceptibility maps:

(1) Collection and extraction of land subsidence conditioning factors: Using the land subsidence areas, we produced a set of land subsidence conditioning factors using ArcGIS. (2) Preparing the training and validation datasets: The dataset was divided into training (70%) and validation datasets (30%). (3) Preparing land subsidence susceptibility models: in this step, we constructed land subsidence susceptibility models using BLR, SVM, LMT and ADTree. (4) Model validation and comparison: all the constructed land subsidence susceptibility models were validated using some statistical indexes including sensitivity, specificity, accuracy, Kappa and RMSE. (5) Production and validation of land subsidence susceptibility maps: the land subsidence susceptibility maps were classified into very low, low, moderate, high and very high susceptibility and maps in ArcGIS. The validation process of the susceptibility maps was checked using ROC, success and prediction rate curves (SR and PR curves) and Friedman and Wilcoxon sign rank tests. Figure 2 shows the flowchart of land subsidence modelling process in this research.

#### 3.1.1. Bayesian Logistic Regression (BLR)

BLR is a combination of logistic regression model and Bayesian method. Compared with classic logistic regression model, BLR can analyze the uncertainties in models by introducing prior distribution and utilizing likelihood function to solve posterior distribution, while over-fit of data may occur in traditional logistic regression model [28]. This BLR consists of three components: (i) determining the prior probability for parameters; (ii) specifying the likelihood function of data; and (iii) estimating the posterior distribution for parameters [29,30]. A Bayesian framework was then comprised to compute the prior probability using land subsidence conditioning factors [31]. Taking Gaussian prior for example, its form is shown as below:

(1) $$l(\beta_{j}|\sigma_{j})=1/(\sqrt{2\pi }\sigma_{j})\exp(-\beta_{j}^{2}/(2\sigma_{j}^{2}))\;$$

where, $\sigma_{j}$ is the standard deviation of Gaussian distribution; and $\beta_{j}$ is the coefficient.

The value of prior variance $\sigma_{j}^{2}$ determines the prior belief of whether $\beta_{j}$ will be near zero (an extremely small value of $\sigma_{j}^{2}$ means $\beta_{j}$ is close to zero). Gaussian prior is related to the *L*2 penalized logistic regression. The Equation (2) needs to be minimized to be subjected to a constraint on the *L*2 norm.

(2) $$L=-l(\beta )+(\lambda /2)\sum_{j=1}^{p}\beta_{j}^{2}\;$$

where, l is log likelihood of data; λ is smoothing parameter that is connected to the standard deviation of Gaussian distribution.

Moreover, for Laplace prior (demonstrated as Equation (3)), we should minimize Equation (4) with *L*1 penalty in accordance to the similar principle of algorithm.

(3) $$l(\beta_{j}|\tau_{j})=\tau_{j}/2\exp(-\tau_{j}\left|\beta_{j}\right|)\;$$

(4) $$L=-l(\beta )+(\lambda /2)\sum_{j=1}^{p}\left|\beta_{j}\right|\;$$

where, $\tau_{j}$ is the prior parameter.

#### 3.1.2. Support Vector Machine (SVM)

SVM as a statistical learning algorithm find an optimal separating hyper plane for classification of class labels [32,33]. SVM was proposed by Vapnik in 1995, which is useful for solving problems around small samples and nonlinearity [34]. For linearly separable samples, all the data can be separated by the optimal separating hyper plane that was searched out using SVM. However, for linearly inseparable samples, in SVM model, all the data should be mapped into a high dimension eigenvector space and then the optimal separating hyper plane can be obtained in the high dimension space. Ultimately, the optimal separating hyper plane can be mapped into original space within a certain error limit. In addition, the selection of kernel functions of SVM model will have a significant impact on results [35,36]. The kernel functions used universally contain several functions: linear (LN), polynomial (PL), radial basis function (RBF) and sigmoid (SIG).

Linear:

(5) $$K(x_{i},x_{j})=x_{i}^{T}\cdot x_{j}\;$$

Polynomial:

(6) $$K(x_{i},x_{j})={\left(\gamma \cdot x_{i}^{T}\cdot x_{j}+r\right)}^{d},\gamma >0\;$$

Radial basis function:

(7) $$K(x_{i},x_{j})=(-\gamma \left\|x_{i}-x_{j}\right\|),\gamma >0\;$$

Sigmoid:

(8) $$K(x_{i},x_{j})=\tanh(\gamma \cdot x_{i}^{T}\cdot x_{j}+r)\;$$

where γ, r and d are parameters of the kernel functions.

#### 3.1.3. Logistic Model Tree (LMT)

LMT is regarded as one of the most stat-of-the-art classifiers in the world [37,38]. LMT is made up of a standard decision tree with logistic regression functions that are built by a logitboost algorithm at the leaves and the process of pruning is implemented by the CART algorithm [39]. The principle of logistic regression function generation was introduced briefly by Karabulut and Ibrikci in 2014 [40]. Initially, a weak classifier is constructed based on the existing sample dataset. Then, some misjudged samples are obtained through repeated application of the weak classifier. In order to emphasize on those misjudged samples, they are given bigger weights. Eventually, several weak classifiers can be compounded into a strong classifier by weighted average method after manifold cycles. In addition, maximum likelihood is employed to find out the specific expressions that linear logistic regression functions need to fit (shown in Equation (9)).

(9) $$F_{c}(x)=a_{0}+\sum_{i}a_{i}x_{i}\;$$

where, $F_{c}(x)$ is the linear logistic regression functions to be fit; $a_{0}$ is the constant term; and $a_{i}$ is the corresponding coefficient of $x_{i}$.

#### 3.1.4. Alternate Decision Tree (ADTree)

ADTree, as one of the most representative data mining methods, is an advanced technique taking root in decision trees and its prediction results are highly accurate [41,42,43]. For this reason, ADTree and other Decision Tree methods have been adopted in the studies on susceptibility assessment [44,45]. In ADTree model, numeric or categorical variables are input generally as the values of a root node. In addition, according to the information gain ratio, the most optimal grouping variables and cut points are figured out. Then, with ADtree growing and pruning, the aim of classifying or predicting the data can be realized. Moreover, ADTree is more appropriate to deal with complex and enormous database due to the boosting technique [46]. Figure 2 shows the flowchart of land subsidence modelling process in the study area.

### 3.2. Factor Selection Using Least Square Support Vector Machine (LSSVM)

The role of each conditioning factor on land subsidence occurrence from one area to another is different due to differences in topography, climate, geology, geomorphology and soil characteristic. At first, we selected all these factors with the assumption that all of them are effective on land subsidence incidence. Then, we selected the ones based on the feature selection techniques such as LSSVM. There are several techniques has been used to quantifying the predictive ability of factors such as Fuzzy-Rough sets [47], Relief [48], Information Gain Ratio [49] and the LSSVM. The LSSVM unlike the IGR which is an entropy-based method that considers only important factors on land subsidence occurrence, assign the weights for all conditioning factors and it does not remove any factors from the modelling process.

In the present study, the least square support vector machine (LSSVM) has been adopted to calculate the importance of each conditioning factor on land subsidence occurrence. LSSVM was proposed by Suykens et al. in (2002) [50]. LSSVM, which is a modified version of SVM, is a kind of statistical kernel based supervised learning methods and benefits from least squares linear as a loss function [51]. These methods analyze data and identify patterns, which are used for classification and regression analysis. LSSVM is completely related to standardization networks [52]. With the quadratic cost function, the optimization problem is reduced to find the solution of a set of linear equations. Given a training set of *N* data points {*xk*, *yk*} *N k* = 1, with input data *xk* ∈ *RN* and output *yk* ∈ *r*, where *RN* and *r* are the *N*-dimensional and the one-dimensional vector space respectively. As the output of the LSSVM method is FS, in this study *x* = [*d*, *c*, *β*, *ϕ*, *ru*, *H*] and *y* = FS. An LSSVM equation model is:

*y*(*x*) = *wTϕ*(*x*) + *b* (10)

where, ϕ (.) is a feature map and prepared the input data into a higher dimensional feature space; *w* ∈ *RN*; *b* ∈ *r*; *w* is an adjustable weight; and *b* is the scalar threshold. For function estimation, optimization problem is formulated as follows:

(11) $$\min imize=0.5*w^{T}w+\gamma \frac{\sum_{k=1}^{n}{e_{k}}^{2}}{2}\;$$

where, *N* is considered as number of data and γ is the regularized parameter that determining the trade-off between the training error minimization and smoothness. 

### 3.3. Evaluation and Comparison of Algorithms

According to Chung and Fabbri (2003) [53], the obtained maps will not be applicable without validation, thus 30% of the data was selected for evaluation as a testing dataset and 70% of the remaining data as a training dataset was applied to model building. In a recent paper, Pham et al. (2016) have stated that the efficiency of performed models should be evaluated and compared for both modeling and testing phases [54]. As the training dataset was used for model building, it only shows the degree of fit; therefore, they cannot be used as model validation criteria. The testing dataset which was not used in the modeling have been applied to model validation. In the current study, three approaches were applied to the evaluation and comparison of the performed model.

#### 3.3.1. Statistical Index Based Evaluation

Several statistical index–based methods, namely sensitivity, specificity, accuracy, Kappa and RMSE were selected to statistically evaluate the performance of the land subsidence models in both training and testing phases. Sensitivity is defined as proportion of land subsidence pixels that correctly classified as land subsidence [54]; specificity is depicted as proportion of non-land subsidence pixels that correctly classified as non-land subsidence; accuracy is the proportion of land subsidence and non-land subsidence pixels that performed models correctly classified, Kappa coefficient was used to evaluate the reliability of the land subsidence models and, RMSE in geosciences is a standard metric for model [30,55].

#### 3.3.2. Receiver Operating Characteristic Curve

Receiver operating characteristic (ROC) curve was used to land subsidence model validation. ROC curve is a standard method and most popular technique to evaluate the quality of the probabilistic models and the area under the curve (AUC) was used to quantitatively validate the models [56]. The ROC curve is prepared using sensitivity and 100-specificity on the *Y* and *X* axes of the diagram. The AUC varies between 0.5–1 as the more the AUC, the higher the prediction capability of the performed model and the higher accurate of the obtained maps [57]. In the current research, both success rate and prediction rate were applied to validate the models.

#### 3.3.3. Statistical Tests of Models

In order to assess whether the performed models are statistically different from each other or not, two inferential statistical models namely the Freidman and Wilcoxon signed rank tests were applied. The Freidman test is a non-parametric test and is used when the data are normally distributed [58]. The null hypothesis to run the Freidman test is that the performances of land subsidence models at significant level of 5% (α = 5%) are not different. The null hypothesis is rejected when the *p*-value is higher than 0.05. The main weakness of the Freidman test is that it only shows whether there is statistically a difference between all performed models or not. To overcome this weakness, the Wilcoxon signed-rank test was performed for pairwise comparison between performances of the land subsidence models. The null hypothesis is similar to the Freidman test but two criteria of *p*-value and *z*-value were applied to evaluate the significance of differences among land subsidence models. If the *p*-value was less than 0.05 and the *z*-value exceeded either −1.96 or +1.96, then the null hypothesis was rejected and this showed that there is a statistically significant difference between the models.

## 4. Results and Discussion

### 4.1. Selection Process of Effective Conditioning Factors on Land Subsidence

**Figure 3:**
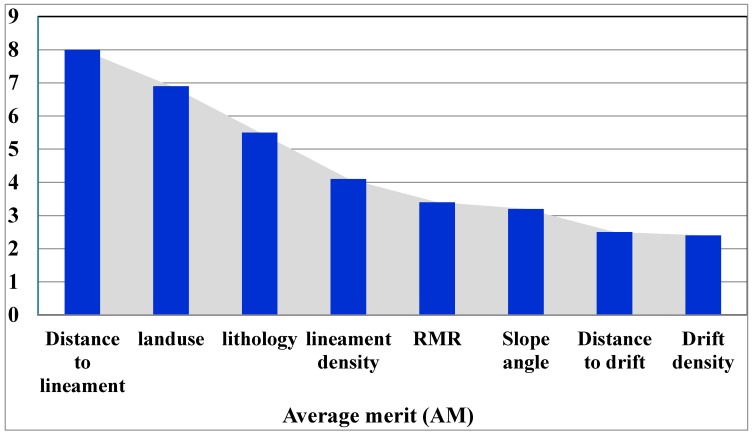
Prediction capability of the most important land subsidence conditioning factors for land subsidence modelling.

Ineffective conditioning factors creates noise and decreases the prediction capability of modelling using training dataset [59]. The results of selecting the most significant conditioning factors affecting land subsidence occurrence are shown in Figure 3. All eight conditioning factors showed significant contribution to the modelling process due to obtaining positive average merit (AM) based on the least square support vector machine (LSSVM) method. The AM is used to prioritize the most important conditioning factors influencing land subsidence modelling. The AM is the average of the LSSVM (Section 2.3) with 10-fold cross-validation. The results revealed that distance to lineament had the highest predictive capability (AM = 8) for land subsidence modelling. It is followed by land use (6.9), lithology (5.5), lineament density (4.1), RMR (3.4), slope angle (3.2), distance to drift (2.5) and drift density (2.4). The results of this study indicated that the distance to lineament is the most important factor for land subsidence occurrence which is in agreement with the finding of Oh et al. (2011) [6]. They reported that the distance to lineament and the distance to drift greatly affected the occurrence of ground subsidence in Jeongahm in Kangwon-do, Korea. Additionally, Saro et al. (2012) [7], after preparing the susceptibility of ground subsidence of Jeongahm in Kangwon-do, Korea, declared that the distance to lineament (faults) was the most significant factor causing land subsidence. On the other word, the faults lead to collapse of the underground coal mine resulting in land subsidence in the study area.

### 4.2. Model Results, Validation and Comparison

The results of model training and validation processes are shown in Table 2. These results have been obtained based on the most effective factors using training dataset (goodness of fit) and validation dataset (performance of models). The results of training and validation processes indicated that all applied machine-learning algorithms have acceptable goodness of fit and predictive capability for spatial prediction of land subsidence in the study area.

Results depicted that the BLR algorithm had the highest sensitivity using training (0.941) and validation (0.714) dataset illustrating that 94.1% and 71.4% of the land subsidence pixels are correctly classified in the land subsidence class. Likewise, the lowest sensitivity was acquired by the ADTree algorithm (training = 0.824; validation, 0.714). In addition, the results of specificity indicated that the BLR algorithm had the highest specificity value (0.882) based on the training phase; while, the BLR and SVM algorithms had the highest value of specificity (0.857) based on the validation dataset. It implies that in the modelling and validation processes, 88.2% and 85.7% of the non-land subsidence pixels were correctly classified with respect to non-land subsidence class. Additionally, the LMT algorithm had the lowest specificity (training = 0.824; validation, 0.714). In terms of accuracy, the BLR algorithm showed the highest value in the modelling (0.912) and validation (0.786) processes. The kappa index for all models varied from 0.764 to 0.822 and 0.428 to 0.571 using training and validation datasets demonstrating a substantial agreement between the models and the reality. The lower the RMSE in the modelling is, the better the performance of results of algorithms will be.

Spatial prediction of land subsidence has rarely been studied using machine learning algorithms. For example, Saro and Park (2013) [5] concluded that the decision tree algorithm had outperformed the frequency ratio approach while Saro et al. (2012) confirmed the obtained results using artificial neural network algorithm [7]. In this study, we compared the results from land subsidence modelling of the BLR as a Bayes-classifier with the SVM as a functional classifier and LMT and ADTree as decision tree classifiers. Results indicated that the ADTree has the lowest power prediction in comparison to other algorithms in the study area. This result is also in agreement with Chen et al. (2017) [60], who demonstrated that the ADTee has the lowest performance in comparison to the kernel logistic regression (KLR) and Naïve Bayes tree (NBTree) for the spatial prediction of landslides. The LMT could better performance and power prediction than the SVM algorithm and less than the BLR algorithm. Wei Chen et al. (2017) [38] concluded that although the LMT had less performance of random forest (RF) algorithm, it had a higher performance than the classification and regression tree (CART) algorithm. Dieu et al. (2014) [59], however, found that the SVM has a high power prediction in comparison to the LMT algorithm. Although SVM is a very universal learner algorithm and ability to learn the dimension ability of the feature space, it is a useful technique for data classification [61]. Some researchers have used SVM as a soft computing benchmark model to assess the power prediction of the new model [57,62]. However, the result of the modelling process encounters some uncertainties including data inputs and the model which used for modelling process. Hence, SVM in some studies has high ability for classification due to less sensitivity and having the higher ability in decreasing over-prediction of susceptible areas which has been observed by other studies [63,64] and while in some other studies has a low prediction in comparison to other algorithms [36,65,66]. On the other hand, BLR which is a combination of logistic regression and Bayes-based theory is a powerful and robust algorithm which has rarely been used in the classification process of landslides [31,67]. In this case, [67] stated some of advantage of the BLR including; (1) BLR parameter estimates are probabilistic estimates or probably distribution rather than the logistic regression, (2) with combining a Bayesian methods with a logistic regression model an alternative to generally used frequent methods and also uncertainty estimation procedures will be better provided resulting in a higher accuracy of parameters estimates. In the current study, the BLR is more powerful and robustness algorithm which could further decreases the noise and over-fitting problems in the modelling process. Therefore, it could well-known as the strong and prominent algorithm in the study area for landslide and non-landslide classifications. Overall, BLR was successfully trained and validated in the modelling and evaluation processes. They were conducted to compute the land subsidence susceptibility indexes for all the pixels in the study area.

### 4.3. Development of Land Subsidence Susceptibility Mapping, Verification and Comparison

Constructing the land subsidence susceptibility mapping (LSSM) with high prediction accuracy depends on the selecting the best parameters of algorithms used for modelling. These parameters are including the number of folds (to reduce error pruning), the number of iterations (to obtain a model with high training and validation accuracy) and the number of seeds (to split the data), C and γ indexes shown in Table 3 for this study. Land subsidence susceptibility indexes (LSSIs) for each pixel of the study area were obtained using the probability distribution function (PDF) of each algorithm individually. It should be noted that the LSSI is the probability of a land subsidence of each pixel over the study area, which ranges between 0 and 1.

**Figure 4:**
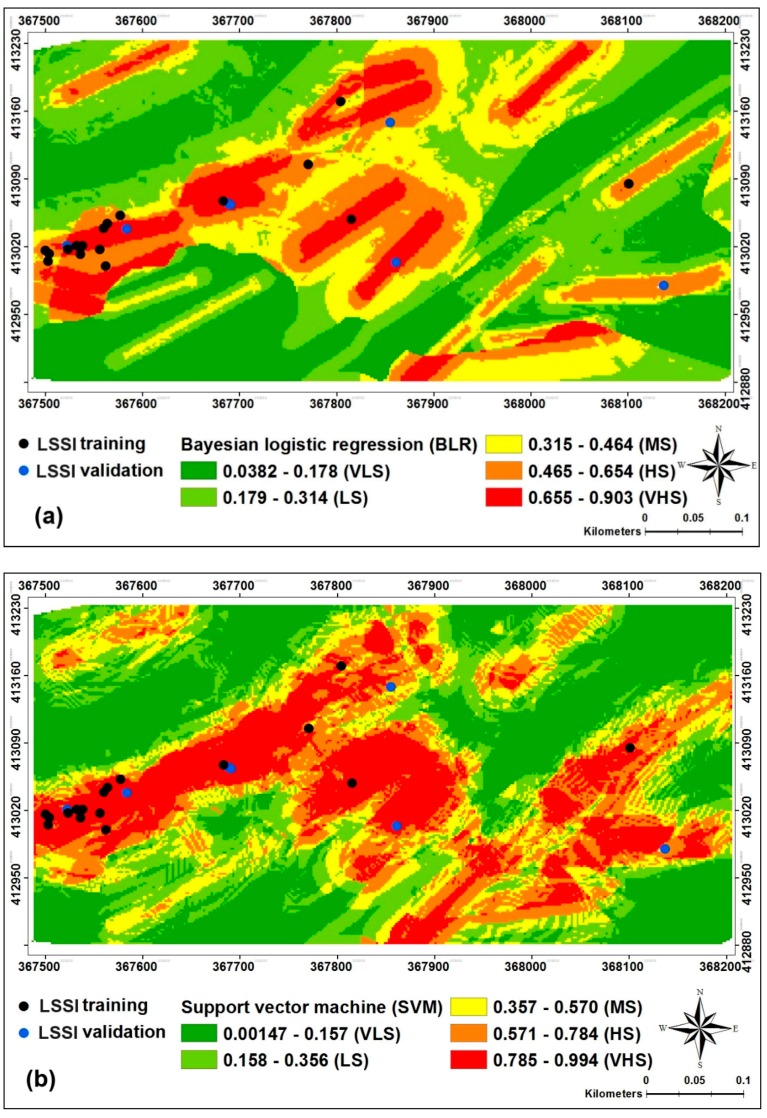

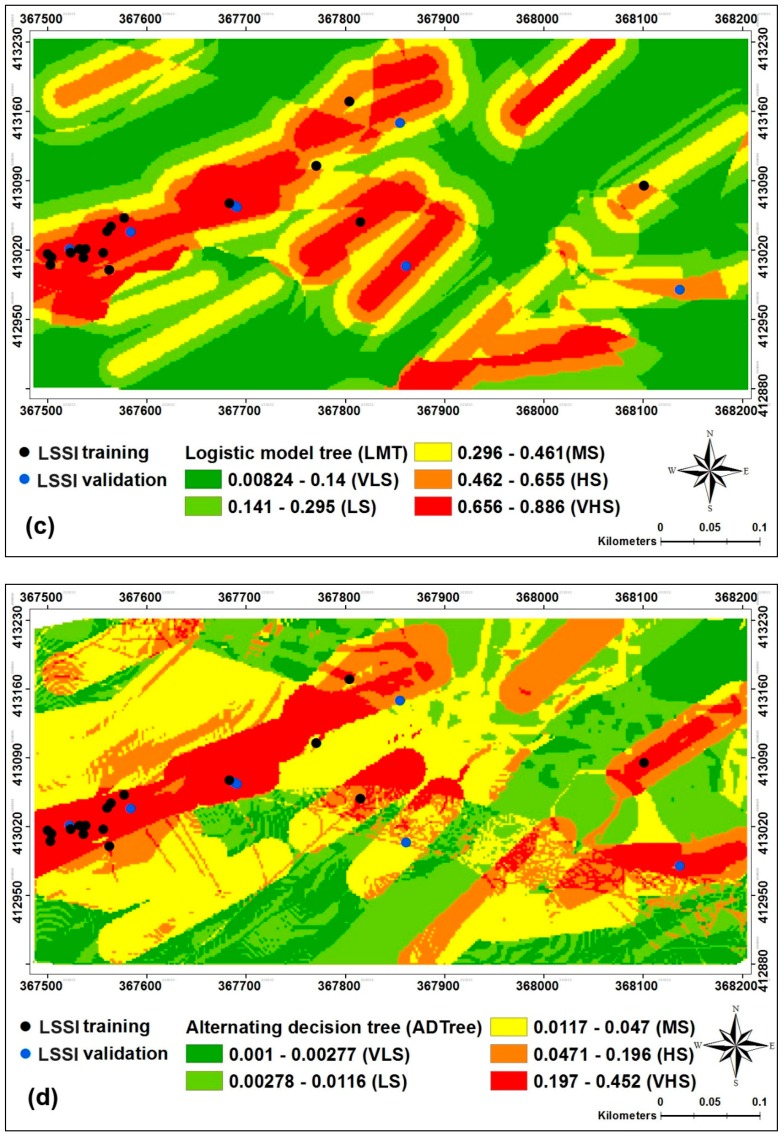
Land subsidence susceptibility maps using: (**a**) the Bayesian logistic regression (BLR), (**b**) the support vector machine (SVM), (**c**) the logistic model tree (LMT) and (**d**) the alternating decision tree (ADTree).

Although there are some techniques for susceptibility map classification in Arc GIS 10.3 software such as natural break, equal interval, geometrical interval, quantile, standard deviations and manual, they should be evaluated and tested to produce a susceptibility map with high conformity with the actual environmental condition [68]. For example, Akgun (2012) has reported that the equal interval or standard deviation classification methods are more proper techniques when the data are close to normal distribution [69]; while the quantile or natural breaks are applicable for the positive or negative skewness of data. Accordingly, in this study, the LSSIs were reclassified using the quantile classification method into five classes including very low susceptibility (VLS), low susceptibility (LS), moderate susceptibility, high susceptibility (HS) and very high susceptibility (VHS) which are shown in Figure 4.

**Figure 5:**
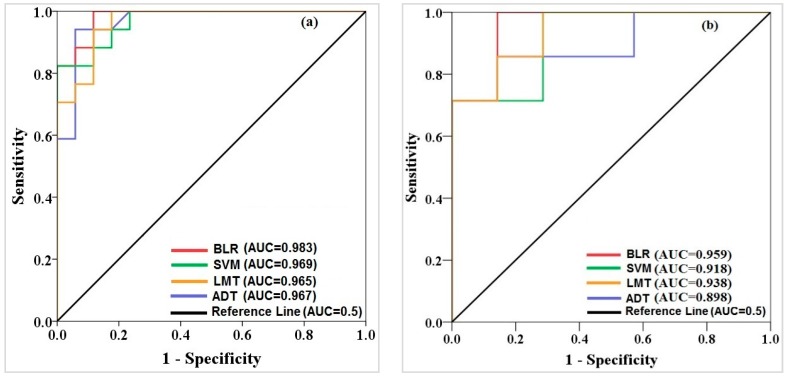
Model validation and comparison using AUROC based on the (**a**) training and (**b**) validation datasets.

The goodness-of-fit (reliability) and prediction accuracy of all machine-learning algorithms have been evaluated using the area under the ROC curve (AUROC) based on the training and validation datasets, respectively. Figure 5 shows the comparison of AUROC for all machine learning algorithms using training (a) and validation (b) datasets. Results of reliability of algorithms concluded that the BLR had the highest value of AUROC in comparison to the SVM (AUROC = 0.969), ADT (AUROC = 0.967) and LMT (AUROC = 0.965) models. Also, results using validation dataset depict that the BLR algorithm had the highest value of the AUROC (0.959), followed by LMT (AUROC = 0.938), SVM (AUROC = 0.918) and ADT (AUROC = 0.898). It pinpoints that BLR had the highest capability in prediction accuracy for modelling of land subsidence compared to the other studied algorithms.

**Figure 6:**
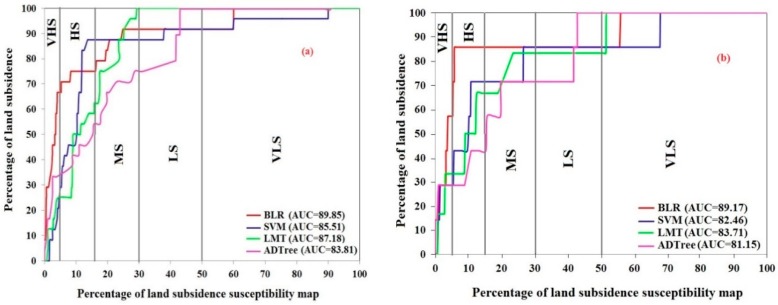
Model validation and comparison using (**a**) success rate curve and (**b**) prediction rate curve.

In addition to the AUROC, we used success rate (SR) and prediction rate (PR) curves to check the reliability and prediction power (accuracy) of all machine-learning algorithms. The difference between the AUROC and SR and PR curves is that in the AUROC, all land subsidence and non-land subsidences locations are applied for training and validation datasets; whereas, only land subsidence locations are used for designing the SR and PR curves. Hence, this difference practically leads to change the values of AUCs. Bui et al. (2016) have reported that because of the lack of corresponding between the AUROC and prediction accuracy of the susceptibility models, the SR and PR curves should be evaluated as well [70]. The results of SR and PR curves are shown in Figure 6. The SR curves of the studied algorithms concluded that the reliability of BLR is higher (AUROC = 0.895), followed by SVM (AUROC = 0.885), LMT (AUROC = 0.871) and ADT (AUROC = 0.838). While, the power prediction of all susceptibility algorithms using the PR curves showed the highest value for BLR (AUROC = 0.891), followed by LMT (AUROC = 0.837), SVM (AUROC = 0.824) and ADT (AUROC = 0.811). It can be noticed that the AUCs calculated by ROC have been slightly lower than those obtained by AUCs of the SR and PR curves. Bui et al. (2016) indicated that these differences are because the ROC curve was plotted based on the entire presence and absent locations, whereas for designing the SR and PR curves used only presence locations for the estimation of area under the curves for all susceptibility maps [70]. They also implied that there is no strict correlation between the AUC of ROC and SR and PR curves. Therefore, the SR and PR should also be considered to check the validity of the susceptibility maps.

Besides AUROC and the SR and PR curves, to further check the applicability of the four machine learning algorithms, the Freidman and Wilcoxon rank tests were used. The aim of these statistical tests is to assess the significant differences between the two or more models. Results of Friedman test illustrated that the values of mean rank for the BLR, the SVM, the LMT and the ADTree algorithms were 1.21, 2.35, 3.56 and 2.88, respectively. Additionally, the chi-square (χ^2^) and statistical significance (Sig.) at 5% confidence interval for all algorithms were obtained as 60.817 and 0.000, respectively.

The results implied that due to having Sig. equals to zero (<0.05), the null hypothesis (no significant difference between the models at the 5% significance level) is rejected and therefor there are statistical differences among all algorithms for land subsidence susceptibility mapping (accepting the zero hypothesis). The Friedman test does not provide any information on statistical differences between two or more algorithms. To compare pairwise algorithms, the Wilcoxon sign ranked test has been used. This test is judged based on the *p*-value and *z*-value criterion so that when the *p*-value < 0.05 and *z*-value > (−1.96 and +1.96), the null hypothesis is rejected and it indicates that the performance of the algorithms to prepare the land subsidence susceptibility maps are significantly different. The result of this test is shown in Table 4. The results clearly concluded that the performances of the all machine learning susceptibility algorithms have statistically significant differences as pairwise. It implies that each algorithm has different results in which in terms of statistically differences there is no evidence of similarity of the results of all algorithms. Therefore, the obtained results from the modelling process based on the statistical assessments can be more reliable and reasonable.

## 5. Conclusions

Land degradation occurs through various surficial features within an area. Land subsidence has been always considered as a degradation process resulting in environmental disasters. Therefore, its identification, assessment, mapping, modelling and management are of crucial importance in any area. The selection of appropriate techniques and models that can provide a clear picture of the system under investigation has been always a challenge while dealing with true world because of its high complexity and big spatial scale. Machine learning algorithms belonging to Data mining approaches have been recently found as appropriate algorithms that are able to assess, model and map different land degradation features around the world with high accuracy. In this study, the land subsidence of Jeong-am area in South Korea were assessed, modelled and mapped using four machine learning algorithms including BLR, SVM, LMT and ADTree through eight conditioning factors. We concluded that if one selects appropriate affecting factors for modelling process, like what happened in this study, machine-learning models can show very high potentials for preparing Land Subsidence Susceptibility Map (LSSM) with highly acceptable accuracy and reliability such that the map can be used as a trusted management tool for degraded areas. The BLR model was distinguished such a model that can assist land managers, conservation authorities, watershed decision-makers and other officials to have a very close look at land subsidence in order to find its best ways of control.

## Figures and Tables

**Table 1:** Land subsidence conditioning factors and their classes.

Land Subsidence Factors	Classes	GIS Data Type	Scale
Slope angle (°)	(1) 0–10; (2) 10–20; (3) 20–30; (4) 30–40; (5) >40	GRID	1 m × 1 m
Distance to drift (m)	(1) 0–2; (2) 2–8; (3) 8–19; (4) 19–50; (5) >50	Line	1:5000
Drift density (m/m^2^)	(1) 0–0.002; (2) 0.002–0.0448; (3) 0.0448–0.120; (4) 0.120–0.299; (5) 0.299–0.952	Polygon	1:5000
Geology	(1) Gobangsan Group; (2) Sadong Group	Polygon	1:50,000
Distance to lineament (m)	(1) 0–10; (2) 10–20; (3) 20–30; (4) 30–60; (5) >60	Line	1:5000
Lineament density (m/m^2^)	(1) 0–0.001; (2) 0.001–0.029; (3) 0.029–0.0435; (4) 0.0435–0.052; (5) 0.052–0.109	Polygon	1:5000
Land use	(1) Mixed forest land; (2) Deciduous forest; (3) Mixed barren land; (4) Commercial area; (5) Coniferous forest; (6) Other grasses; (7) Transportation; (8) Natural grasses; (9) Field	Polygon	1:50,000
RMR	(1) 0.00366–1.26; (2) 1.26–1.54; (3) 1.54–1.93; (4) 1.93–2.79; (5) 2.79–4	Polygon	1:5000

**Table 2:** Model results and analysis using training and validation datasets. TP: true positive, TN: true negative, FP: false positive, FN: false negative, SST: sensitivity, SPC: specificity, ACC: accuracy, T: training; V: validation.

	BLR		SVM		LMT		ADTree	
	T	V	T	V	T	V	T	V
TP	16	5	16	4	15	5	14	4
TN	15	6	14	6	14	5	15	5
FP	2	1	2	1	3	2	2	2
FN	1	2	3	3	2	2	3	3
SST	0.941	0.714	0.842	0.571	0.882	0.714	0.824	0.571
SPC	0.882	0.857	0.875	0.857	0.824	0.714	0.882	0.714
ACC	0.912	0.786	0.857	0.714	0.853	0.714	0.853	0.643
Kappa	0.822	0.571	0.764	0.571	0.764	0.428	0.764	0.428
RMSE	0.297	0.426	0.323	0.430	0.335	0.432	0.363	0.462

**Table 3:** Parameters of machine learning algorithms applied in this study.

Algorithm	Parameters
BLR	Hyper parameter value range, R: 0.01–3.16; Specific hyper parameter value, 0.27; The maximum number of iterations to perform, 1000; The number of folds in the internal cross-validation or pruning, 2; The random number seed, 1; the threshold for classification, 0.5.
LMT	The minimum number of instances at which a node is considered for splitting, 15; a fixed number of iterations for LogitBoost, −1.
SVM	Build logistic model, False; C, 0.1; epsilon, 1.0 × 10^−12^; filter type, normalized training data; kernel function, polykernel; number of folds, −1; random seed, 1; tolerance parameter, 0.001.
ADT	Number of boosting iteration, 10; random seed, 0; search path, expand all paths

**Table 4:** Performance comparison of the machine learning models in land subsidence using Wilcoxon signed-rank test (two-tailed). The standard *p*-value is 0.05.

No.	Pair Wise Comparison	Number of Positive Differences	Number of Negative Differences	*z*-Value	*p*-Value	Significance
1	BLR vs. SVM	27	7	−4.078	0.000	Yes
2	BLR vs. LMT	24	10	−2.522	0.012	Yes
3	BLR vs. ADTree	28	4	−4.469	0.000	Yes
4	SVM vs. LMT	27	7	−4.043	0.000	Yes
5	SVM vs. ADTree	33	1	−5.069	0.000	Yes
6	LMT vs. ADTree	33	1	−5.003	0.000	Yes
